# The clinical prediction model to distinguish between colonization and infection by *Klebsiella pneumoniae*

**DOI:** 10.3389/fmicb.2024.1508030

**Published:** 2025-01-23

**Authors:** Xiaoyu Zhang, Xifan Zhang, Deng Zhang, Jing Xu, Jingping Zhang, Xin Zhang

**Affiliations:** ^1^First Department of Infectious Diseases, The First Affiliated Hospital of China Medical University, Shenyang, China; ^2^Department of Infectious Diseases, The First Affiliated Hospital of Xiamen University, Xiamen, China

**Keywords:** *Klebsiella pneumoniae*, colonization, infection, sputum culture, risk factors, machine learning, clinical prediction model

## Abstract

**Objective:**

To develop a machine learning-based prediction model to assist clinicians in accurately determining whether the detection of *Klebsiella pneumoniae* (KP) in sputum samples indicates an infection, facilitating timely diagnosis and treatment.

**Research methods:**

A retrospective analysis was conducted on 8,318 patients with KP cultures admitted to a tertiary hospital in Northeast China from January 2019 to December 2023. After excluding duplicates, other specimen types, cases with substandard specimen quality, and mixed infections, 286 cases with sputum cultures yielding only KP were included, comprising 67 cases in the colonization group and 219 cases in the infection group. Antimicrobial susceptibility testing was performed on the included strains, and through univariate logistic regression analysis, 15 key influencing factors were identified, including: age > 62 years, ESBL, CRKP, number of positive sputum cultures for KP, history of tracheostomy, use of mechanical ventilation for >96 h, indwelling gastric tube, history of craniotomy, recent local glucocorticoid application, altered consciousness, bedridden state, diagnosed with respiratory infectious disease upon admission, electrolyte disorder, hypoalbuminemia, and admission to ICU (all *p* < 0.05). These factors were used to construct the model, which was evaluated using accuracy, precision, recall, F1 score, AUC value, and Brier score.

**Results:**

Antimicrobial susceptibility testing indicated that the resistance rates for penicillins, cephalosporins, carbapenems, and quinolones were significantly higher in the infection group compared to the colonization group (all *p* < 0.05). Six predictive models were constructed using 15 key influencing factors, including Classification and Regression Trees (CART), C5.0, Gradient Boosting Machines (GBM), Support Vector Machines (SVM), Random Forest (RF), and Nomogram. The Random Forest model performed best among all indicators (accuracy 0.93, precision 0.98, Brier Score 0.06, recall 0.72, F1 Score 0.83, AUC 0.99). The importance of each factor was demonstrated using mean decrease in Gini. “Admitted with a diagnosis of respiratory infectious disease” (8.39) was identified as the most important factor in the model, followed by “Hypoalbuminemia” (7.83), then “ESBL” (7.06), “Electrolyte Imbalance” (5.81), “Age > 62 years” (5.24), “The number of Positive Sputum Cultures for KP > 2” (4.77), and being bedridden (4.24). Additionally, invasive procedures (such as history of tracheostomy, use of ventilators for >96 h, and craniotomy) were also significant predictive factors. The Nomogram indicated that CRKP, presence of a nasogastric tube, admission to the ICU, and history of tracheostomy were important factors in determining KP colonization.

**Conclusion:**

The Random Forest model effectively distinguishes between infection and colonization status of KP, while the Nomogram visually presents the predictive value of various factors, providing clinicians with a reference for formulating treatment plans. In the future, the accuracy of infection diagnosis can be further enhanced through artificial intelligence technology to optimize treatment strategies, thereby improving patient prognosis and reducing healthcare burdens.

## 1 Introduction

**Figure 1:**
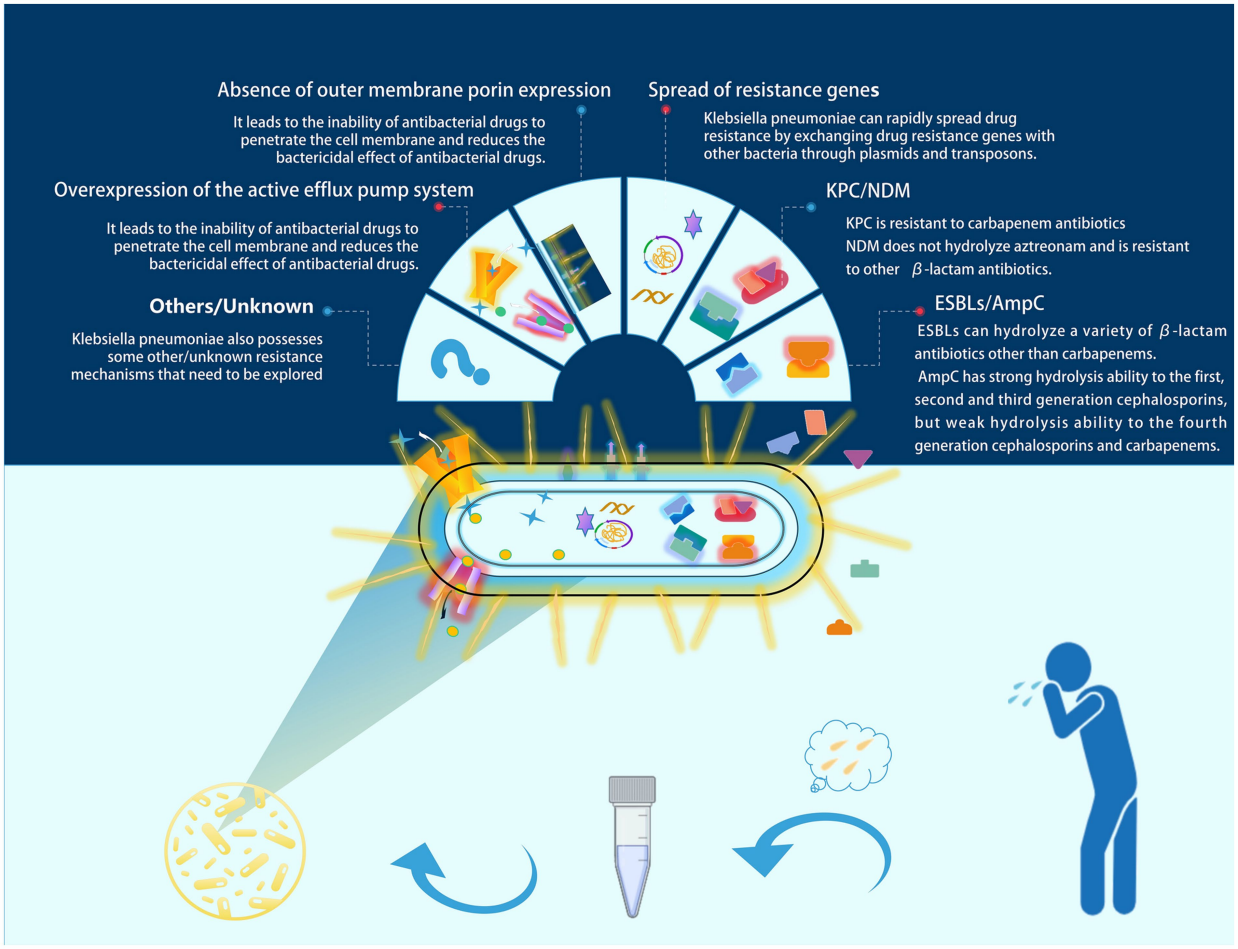
Major resistance mechanisms of KP. In clinical practice, sputum culture is an important tool for diagnosing respiratory tract infections. However, misinterpreting colonizing *Klebsiella pneumoniae* as an infectious pathogen may lead to unnecessary antimicrobial treatment. In such cases, this could contribute to the development of resistance in *Klebsiella pneumoniae*. We have illustrated several common resistance mechanisms of *Klebsiella pneumoniae* in the figure.

*Klebsiella pneumoniae* (commonly referred to as KP) is a frequently encountered opportunistic pathogen in clinical settings, typically colonizing the human gut and upper respiratory tract (Podschun and Ullmann, 1998). KP colonization can be detected in more than 40% of the population (Guilhen et al., 2019). At the same time, this bacterium is the third most common bacterial cause of hospital-acquired pneumonia (Jones, 2010). KP infections are also common in ventilator-associated pneumonia (VAP) (Alnimr, 2023). With the emergence of multi-drug resistant (MDR) strains, it has been classified by the World Health Organization (WHO) as a priority pathogen for which urgent new therapies are needed (Antimicrobial resistance: global report on surveillance, n.d.). Sputum culture is an important method for identifying respiratory tract infection pathogens in clinical practice; however, for the opportunistic pathogen KP, clinicians often find it challenging to distinguish between colonization and infection. Colonization refers to the adherence and growth of microorganisms on the surface of host tissues, while infection describes the process by which microorganisms invade host cells or tissues, leading to pathological conditions. Colonization and infection are closely related (Vornhagen et al., 2024). When the host’s immune response is suppressed or there are other risk factors present, colonizing *Klebsiella pneumoniae* may begin to proliferate and invade surrounding tissues, leading to severe infection (Cai et al., 2024). Misclassifying colonization as infection can lead to overtreatment in clinical settings, increasing the incidence of adverse reactions and promoting the development of antibiotic resistance. This includes mechanisms such as enzymatic antibiotic inactivation and modification (e.g., ESBLs, AmpC, and NDM), spread of resistance genes, absence of outer membrane porin expression, and overexpression of active efflux pump systems, among others (Li et al., 2023) (as shown in Figure 1, which illustrates the main resistance mechanisms of KP). Conversely, misclassifying infection as colonization can delay clinical treatment and increase patient mortality rates. Therefore, there is a need for a tool to assist clinicians in better differentiating between KP colonization and infection.

Previous studies have typically used logistic regression analysis to identify risk factors associated with infection and colonization (Wang X. et al., 2024). However, logistic regression struggles to capture complex nonlinear relationships between features and lacks intuitive clarity. In recent years, artificial intelligence and machine learning have shown great potential in the diagnosis and treatment of bacterial infections. They can effectively handle complex data, integrate various patient characteristics to construct predictive models, and help doctors understand more intuitively how different clinical features influence the occurrence of diseases, providing more reliable support for clinical decision-making (Deo, 2015; Jiang et al., 2022; Seyer Cagatan et al., 2022). Therefore, the aim of this study is to develop a predictive model that can accurately identify colonization versus infection of KP in sputum cultures. This model is intended to provide clinicians with scientific evidence to optimize treatment strategies and reduce the risk of misdiagnosis and inappropriate use of antimicrobial agents.

## 2 Methods

### 2.1 Setting and participants

**Figure 2:**
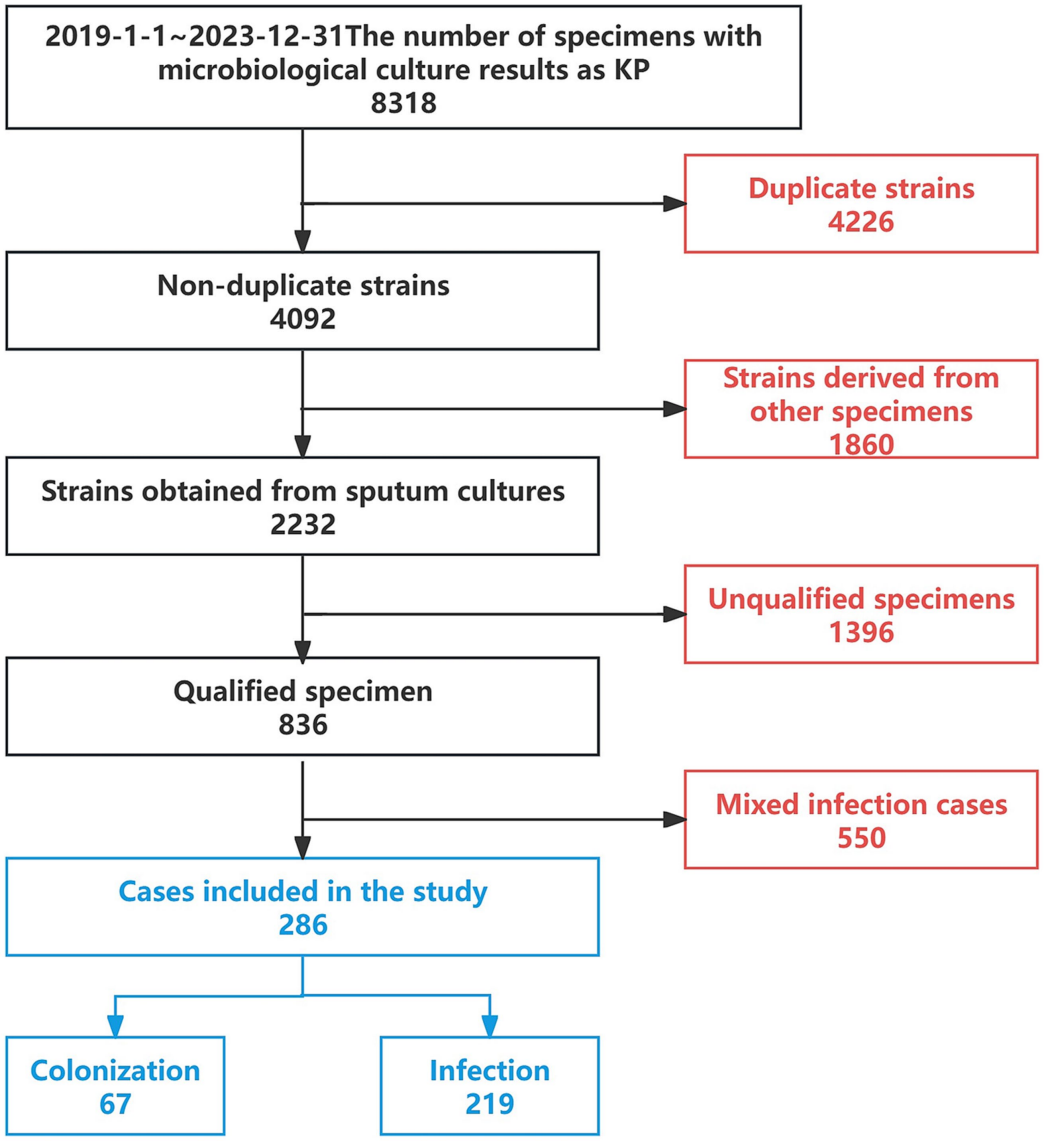
Case selection process.

**Figure 3:**
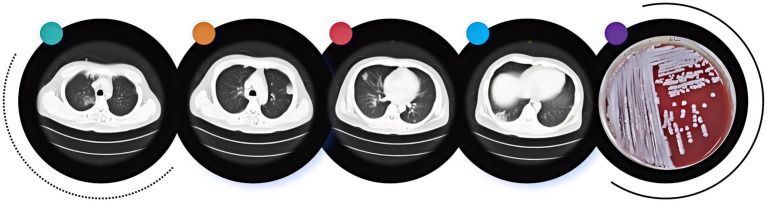
CT imaging and sputum culture of a typical case of pneumonia due to KP.

A retrospective analysis was conducted on 8,318 patients with microbiological cultures identified as KP admitted to a tertiary hospital in Northeast China from January 2019 to December 2023. After excluding duplicate cases, other types of specimens, cases with substandard specimen quality, and mixed infections, a total of 286 cases were included, which consisted of patients with sputum cultures yielding only *Klebsiella pneumoniae*. Among these, 67 cases were classified as the colonization group and 219 cases as the infection group (Figure 2). This study has been reviewed and approved by the Ethics Committee of the First Affiliated Hospital of China Medical University [Ethics ID(2023)2023-142-2]. Infectious disease specialists confirmed the diagnosis of KP infection or colonization (diagnostic criteria are shown in Table 1). (In Figure 3 we show the CT lung images and sputum cultures of a relatively typical case of pneumonia due to KP).

**Table 1:** Inclusion, exclusion, and diagnostic criteria.

**Inclusion criteria**	**Diagnostic criteria**
Sputum culture positive for KP Sample quality meets standards	The diagnosis of KP infection or colonization was confirmed by infectious disease specialists based on the “Diagnostic Criteria for Hospital-Acquired Pneumonia and Ventilator-Associated Pneumonia in Chinese Adults” (2018 Edition) (Subspecialty Group of Infectious Diseases, Respiratory Society, Chinese Medical Association, 2018) and the “Guidelines for the Diagnosis and Treatment of Adult Community-Acquired Pneumonia” (Practical Edition, 2018) (Chinese Medical Association, Chinese Medical Association Journal, Chinese Medical Association General Medicine Society et al., 2019).
**Duplicate strains**	
Non-sputum samples Samples of unsatisfactory quality Cases with mixed viral, other bacterial, or fungal infections	

### 2.2 Microbiological methods

Clinical data for the included cases were collected through the hospital’s electronic medical record system. Using a case–control study design, a retrospective analysis of the clinical data from all patients was conducted. Antimicrobial susceptibility testing was performed on the KP (KP) strains obtained from the included cases. Identification of all pathogens and antimicrobial susceptibility testing were carried out using the VITEK 2 automated bacterial identification and VITEK 2 Compact antimicrobial susceptibility testing system from bioMérieux, France. The results of the antimicrobial susceptibility tests were categorized as susceptible, intermediate, and resistant according to the standards set by the Clinical and Laboratory Standards Institute (CLSI) (CLSI, 2023; Vornhagen et al., 2024). Extended spectrum beta-lactamase (ESBL) strains were detected through double disk synergy tests and phenotypic confirmation tests (antimicrobial susceptibility test disks were purchased from Oxoid, United Kingdom). In this study, carbapenem-resistant bacteria were defined as those resistant to either imipenem or meropenem.

### 2.3 Statistical method

Statistical analysis was conducted using SPSS 27.0 software. Categorical data were expressed as counts and percentages. The Chi-square test was used to compare the differences in resistance rates between the KP colonization group and the infection group (*p* < 0.05 was considered statistically significant). Univariate binary logistic regression analysis was performed to identify factors with statistically significant differences between the two groups (p < 0.05 was considered statistically significant), and these factors were included as predictive variables in the model. Predictive models to distinguish between KP colonization and infection were constructed using R 4.4.1 software, including Classification and Regression Trees (CART), C5.0, Gradient Boosting Machine (GBM), Support Vector Machine (SVM), Random Forest (RF), and Nomogram models. The function set.seed(42) was used to ensure the reproducibility of the random process, allowing the same data to yield identical training and test set divisions. The trainControl function was employed to set the model training control parameters for 10-fold cross-validation. The performance of each model was evaluated using accuracy, precision, recall, F1 Score, AUC value, and Brier score.

## 3 Results

### 3.1 Antimicrobial susceptibility testing of KP

**Figure 4:**
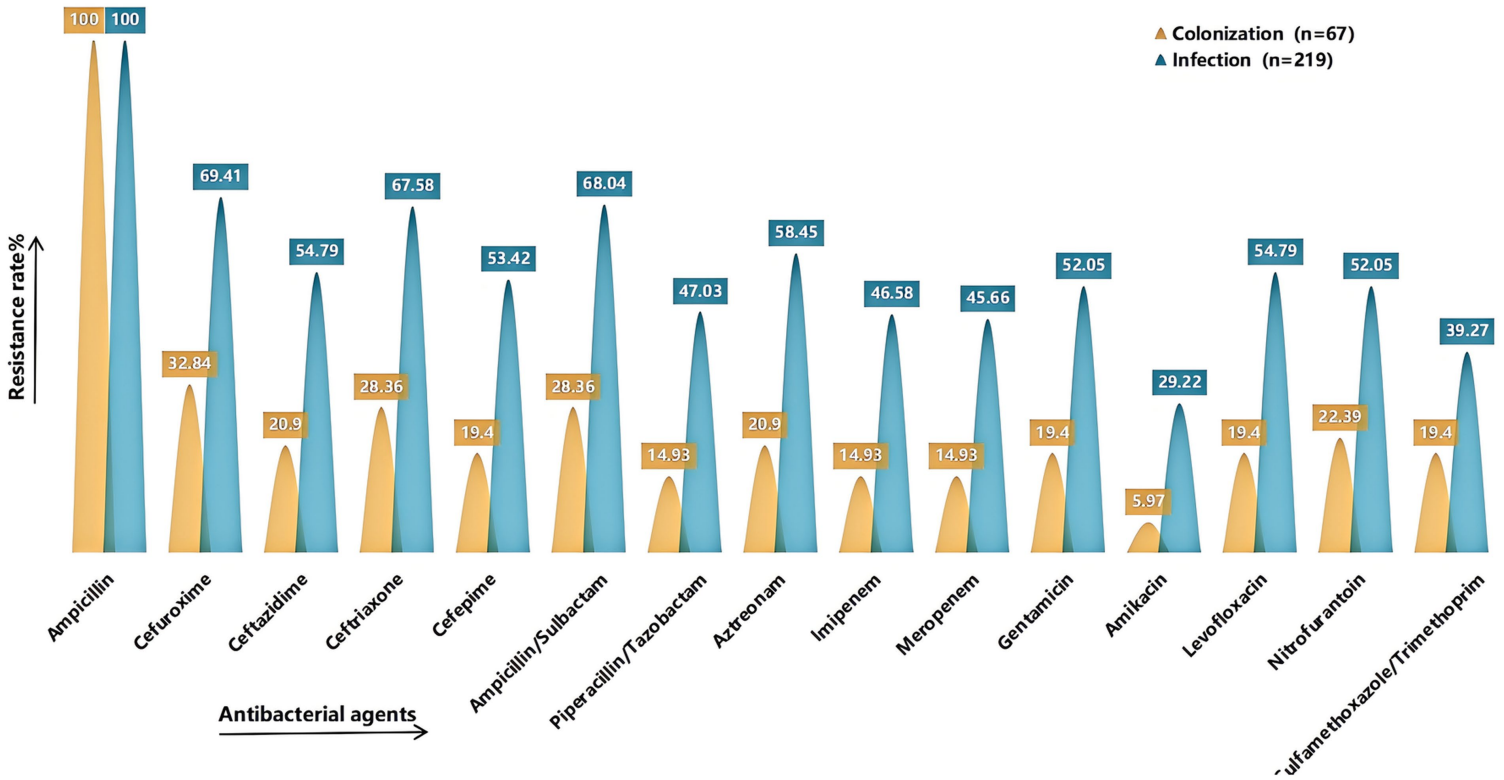
Comparison of drug resistance rates. It shows the rates of resistance to common antimicrobial agents in the colonized and infected groups. The X-axis represents the antimicrobial agents (Ampicillin, Cefuroxime, Ceftazidime, Ceftriaxone, Cefepime, Ampicillin/Sulbactam, Piperacillin/Tazobactam, Aztreonam, Imipenem, Meropenem, Gentamicin, Amikacin, Levofloxacin, Nitrofurantoin, Sulfamethoxazole/Trimethoprim), and the Y-axis represents the resistance rate. Yellow indicates the colonized group and green indicates the infected group. Detailed information can be found in Supplementary Table S1.

Antimicrobial susceptibility testing was performed on strains isolated from sputum cultures of the final 286 enrolled cases (Figure 4). Compared with the colonization group, the infection group exhibited generally higher resistance rates to various antibiotics, with all differences being statistically significant (*p* < 0.05) (see Supplementary Table S1 for detailed results). A total of 141 CRKP strains were isolated, including 17 strains from the colonization group and 124 strains from the infection group.

### 3.2 Clinical characteristics of the participants

The clinical data of the included cases were collected (Table 2), and univariate logistic regression analysis revealed the following important factors influencing colonization and infection: age > 62 years, ESBL positivity, CRKP positivity, number of positive sputum cultures for KP, history of tracheostomy, use of ventilator for >96 h, presence of indwelling gastric tube, craniotomy, recent history of topical glucocorticoid use, altered consciousness, bedridden status, diagnosis of respiratory infectious disease upon admission, electrolyte disturbances, low serum protein levels, and admission to ICU (all *p* < 0.05).

**Table 2:** Clinical characteristics of cases with KP colonization and infection and univariate logistic regression analysis.

Clinical characteristics/influencing factors			Colonization [*n* = 67, Strains(%)]	Infection [*n* = 219, Strains(%)]	Univariate logistic regression analysis	
					*p*	95%CI
Basic information	Sex	Male	53(79.10)	163(74.43)	0.437	0.396–1.491
		Female	14(20.90)	56(25.57)		
	Age^a^	>62	26(38.8)	124(56.62)	0.011	1.176–3.601
	ESBL		19(28.36)	149(68.04)	<0.01	2.944–9.822
	CRKP		17(25.37)	124(56.62)	<0.01	2.082–7.078
	The number of Positive Sputum Cultures for KP > 2^b^		5(7.46)	81(36.99)	<0.01	2.810–18.849
Underlying disease	Smoking for ≥ 10 Years		15(22.39)	46(21.00)	0.809	0.476–1.784
	Hypertension		17(25.37)	64(29.22)	0.541	0.652–2.263
	Diabetes		15(22.39)	61(27.85)	0.782	0.702–2.553
	Coronary heart disease		9(13.43)	20(9.13)	0.310	0.280–1.499
	Cerebrovascular disease		11(16.42)	62(28.31)	0.054	0.988–4.090
	Gastrointestinal bleeding		2(2.99)	10(4.57)	0.575	0.332–7.279
Invasive procedure	Tracheal intubation		31(46.27)	110(50.23)	0.571	0.677–2.028
	Tracheostomy		11(16.42)	65(29.68)	0.034	1.058–4.364
	Ventilator		32(47.76)	132(60.27)	0.071	0.957–2.878
	Ventilator use > 96 h		11(16.42)	85(38.81)	0.01	1.602–6.511
	Bronchoscope		7(10.45)	27(12.33)	0.678	0.500–2.907
	Peripherally inserted central catheter		20(29.85)	73(33.33)	0.595	0.649–2.128
	Gastric tube indwelling		31(46.27)	137(62.56)	0.019	1.116–3.372
	Recent surgery		25(37.31)	70(31.96)	0.416	0.446–1.397
	Craniotomy		4(5.97)	36(16.44)	0.039	1.061–9.050
Medication history in the last 2 weeks	History of proton pump inhibitor (PPI) use		38(56.72)	148(67.58)	0.104	0.909–2.785
	History of systemic steroid use		12(17.91)	73(33.33)	0.18	1.155–4.545
	Recent history of topical glucocorticoid use^c^		19(28.36)	98(44.75)	0.018	1.129–3.707
Others	Dysbiosis		12(17.91)	50(22.83)	0.394	0.674–2.730
	Disturbance of consciousness		10(14.93)	78(35.62)	0.002	1.525–6.521
	Bedridden status		43(64.18)	190(86.76)	<0.01	1.940–6.894
	Admitted with a diagnosis of respiratory infectious disease		13(19.40)	97(44.29)	<0.01	1.704–6.40
	Moderate to severe anemia		13(19.40)	51(23.29)	0.505	0.638–2.493
	Electrolyte imbalance		27(40.30)	144(65.75)	<0.01	1.621–4.991
	Hypoalbuminemia		22(32.84)	149(68.04)	<0.01	2.429–7.805
	Liver dysfunction		24(35.82)	107(48.86)	0.062	0.973–3.013
	Kidney dysfunction		8(11.94)	37(16.89)	0.332	0.661–3.400
	Heart Failure		4(5.97)	20(9.13)	0.418	0.522–4.804
	Admitted to the ICU		32(47.76)	145(66.21)	0.007	1.23–3.734
Prognosis	Die		7(10.45)	40(18.26)	0.136	0.815–4.502

a The average age of the subjects in this study is 62 years, which is used as the cutoff value.

b The number of KP sputum cultures: the average number of KP sputum cultures in all cases was 2, which was the critical value.

c Recent history of topical glucocorticoid use was defined as nebulized glucocorticoid inhalation.

### 3.3 Construction and evaluation of predictive models

The 15 important influencing factors obtained from univariate logistic regression analysis were used to construct models including Classification and Regression Trees (CART), C5.0, Gradient Boosting Machine (GBM), Support Vector Machine (SVM), Random Forest (RF), and Nomogram. These models were designed to differentiate between colonization and infection status of KP. The performance of the models was evaluated using several metrics, including accuracy, precision, recall, F1 Score, Area Under the Curve (AUC), and Brier Score.

**Figure 5:**
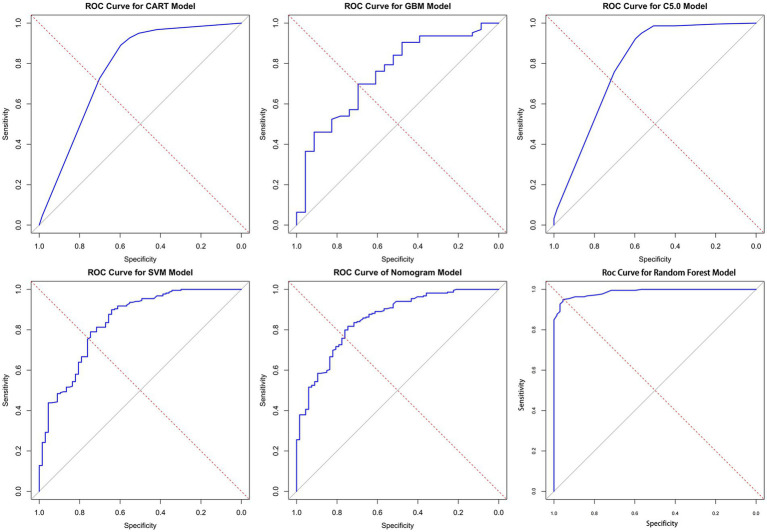
ROC curves for the six models. Here are the ROC (receiver operating characteristic) curves for six different models, with the horizontal axis of each plot representing specificity, the vertical axis representing sensitivity, and the diagonal as the baseline for random guesses. AUC is an important indicator of ROC curve, which is used to evaluate the classification performance of the model. The values of AUC range from 0 to 1 and are explained as follows: AUC = 1: perfect. 0.7 ≤ AUC < 0.8: good. 0.6 ≤ AUC < 0.7: general. AUC < 0.6 was considered poor. In the figure, the closer the curve is to the upper left corner, the higher the AUC value, indicating better model performance (the specific AUC values can be found in Table 3).

In this study, the predictive models were evaluated, and the Random Forest model demonstrated the best performance across all metrics: accuracy of 0.93, precision of 0.98, Brier Score of 0.06, recall of 0.72, and F1 Score of 0.83, indicating excellent predictive performance. The C5 model also performed well, with an accuracy of 0.87, precision of 0.92, and an F1 Score of 0.65, suggesting its reliability in predicting KP infections. The CART model achieved an accuracy of 0.85, precision of 0.76, and an F1 Score of 0.61, showing performance similar to that of the C5 model. Although both C5 and CART effectively distinguished between colonization and infection, their recall rates were relatively low, and their Brier Scores were higher, indicating slightly insufficient predictive performance. The SVM model had an accuracy of 0.84 and effectively identified infection samples, reducing the risk of false negatives; however, its overall performance was still inferior to that of the C5 and CART models. The GBM model performed relatively weakly across all metrics, particularly in recall (0.35) and F1 Score (0.46), suggesting a higher probability of missed detections. The AUC (Area Under the Curve) is an important metric for measuring a model’s discriminative ability. In this study, the Random Forest model achieved an AUC of 0.99, while the Nomogram model also exhibited a high AUC of 0.85. In contrast, the AUC values for the CART, GBM, and C5 models ranged from 0.75 to 0.80, indicating moderate discrimination ability. In summary, the Random Forest model performed best in distinguishing between colonization and infection of KP, while the Nomogram model also proved to be a reliable choice, suitable for the accurate identification of colonization and infection states in clinical applications (see Figure 5 and Table 3).

**Table 3:** Evaluation measures of the model.

	CART	GBM	C5	SVM	Nomogram	Random forest
Accuracy	0.85	0.78	0.87	0.84	0.84	0.93
Precision	0.76	0.67	0.92	0.73	0.72	0.98
Recall	0.51	0.35	0.51	0.49	0.51	0.72
F1 score	0.61	0.46	0.65	0.59	0.60	0.83
AUC	0.77	0.75	0.80	0.84	0.85	0.99
Brier score	0.12	0.17	0.11	6.56	0.12	0.06

### 3.4 Evaluation of feature importance in the Random Forest model

**Figure 6:**
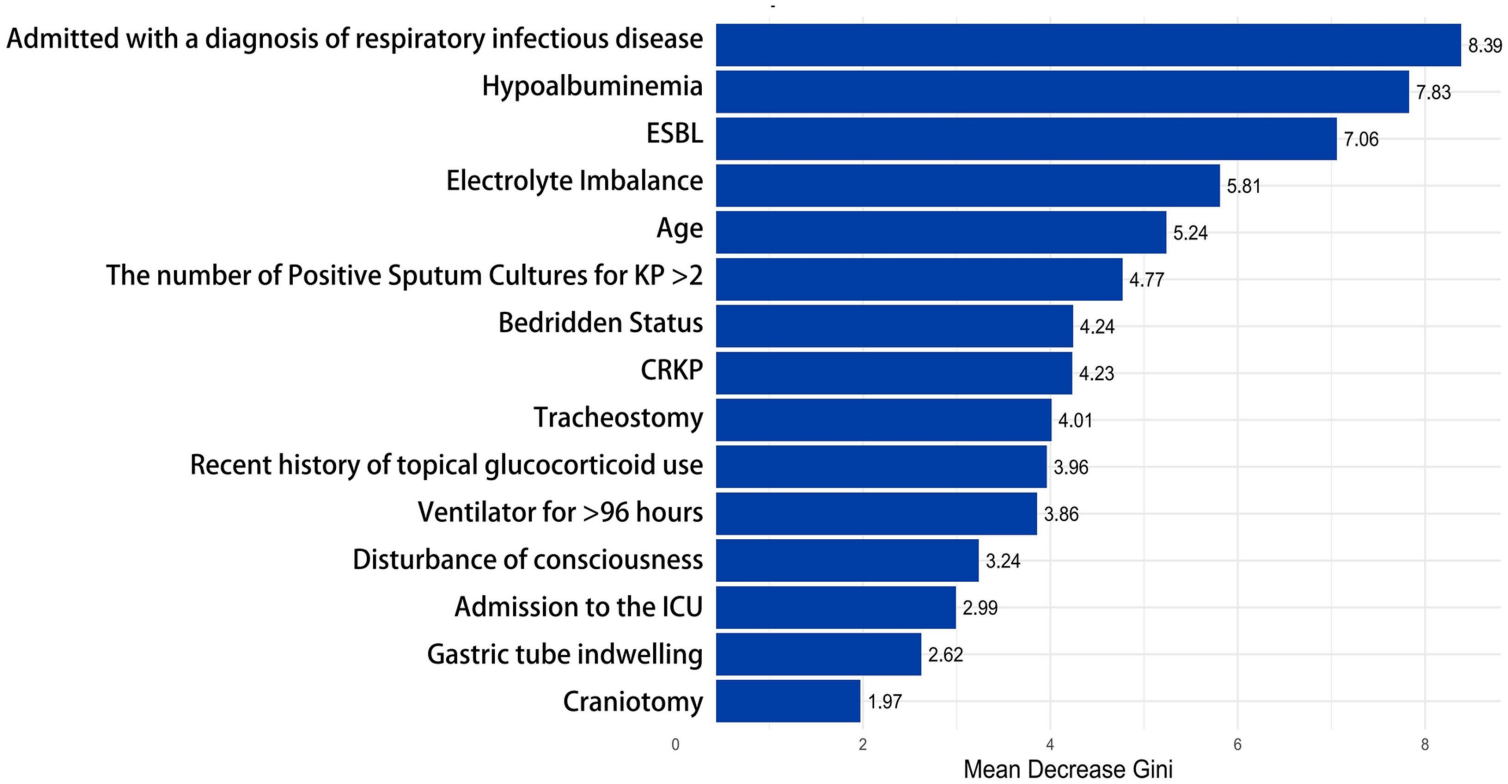
Evaluation of feature importance in the random forest model. This bar chart shows the importance of different clinical factors in the random forest model. The X-axis represents the “Mean Decrease Gini,” while the Y-axis lists the clinical factors, arranged in order of their importance to the outcome from high to low. ≥ 8.0, High; 5.0–7.9, Medium-High; 3.0–4.9, Medium; 1.0–2.9, Low; 0.0–0.9, Very Low or None.

To better understand the relationship between the model and the data, we visually analyzed the best-performing Random Forest model using Mean Decrease Gini. The X-axis represents the “Mean Decrease Accuracy” values for each feature; higher values indicate a more significant decline in model performance when that feature is removed. The influencing factors are ranked according to their importance, with features at the top contributing the most to the model. The most important factor in the model is “Admitted with a diagnosis of respiratory infectious disease” (8.39). The second most important feature is “Hypoalbuminemia” (7.83), followed by “ESBL” (7.06), “Electrolyte Imbalance” (5.81), “Age” (5.24), and “The number of Positive Sputum Cultures for KP > 2” (4.77). In contrast, factors such as “Craniotomy” (1.97), “Nasogastric Tube Placement” (2.62), and “ICU Admission” (2.99) contributed relatively little to determining whether the sputum culture for KP indicated infection (see Figure 6).

### 3.5 Nomogram model

**Figure 7:**
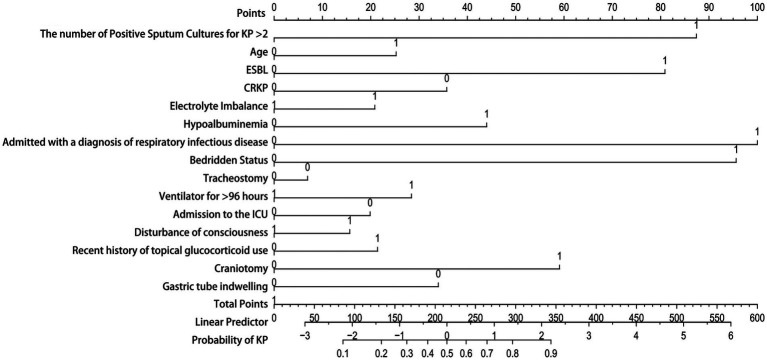
Nomogram model. The Y-axis of the nomogram represents various clinical factors, with each axis line corresponding to an input variable. The figure 0 on the axis indicates colonization, while the figure 1 indicates infection. The scale value on the X-axis represents the points or measurements of that clinical factors, and the corresponding point can be found to determine the contribution of that factor in the total score.

Due to the good performance of the Nomogram, which is more intuitive than the Random Forest model, we also presented the drawn Nomogram (Figure 7). It clearly shows that “Admitted with a diagnosis of respiratory infectious disease” is the most important factor, followed by “bedridden status,” “the number of positive sputum cultures for KP > 2,” ESBL, and craniotomy. Additionally, CRKP, nasogastric tube placement, ICU admission, and tracheostomy are all important factors for determining KP colonization.

## 4 Discussion

In this study, the resistance rates of various antimicrobial drugs in the infection group were generally higher than those in the colonization group, with statistically significant differences (*p* < 0.05). The proportion of ESBL and CRKP in the infection group was significantly higher than that in the colonization group, indicating that the infection status is highly likely associated with the extensive use of antimicrobial drugs. Previous studies have shown that the use of broad-spectrum antimicrobial agents and carbapenems are independent risk factors for the occurrence of ESBL and CRKP (Lou et al., 2022; Zhu et al., 2023; Lopera et al., 2024). High resistance rates will place significant pressure on the selection of antimicrobial agents in clinical practice. Infection status not only increases the demand for antimicrobial drugs among patients but may also accelerate the proliferation and transmission of resistant strains. Previous studies have indicated that colonizing strains of KP (KP) have a high degree of homology with infecting isolates, with 50% of KP infections arising from the patient’s own microbiota (Gorrie et al., 2017). However, this study found that there was a significant difference in the resistance rates of the strains isolated from the colonization group and the infection group. From the host’s perspective (Gonzalez-Ferrer et al., 2021), during an infection, the patient’s immune system may be compromised, and the transmission of resistant strains through cross-infection in healthcare environments, such as hospitals (Córdova-Espinoza et al., 2023), may result in the strains in the infection group being more resistant. In contrast, the strains in the colonization group may not experience a significant increase in resistance due to the pressure from antimicrobial agents, as they exist in a relatively stable colonization state. From a microbiological perspective (Gomez-Simmonds and Uhlemann, 2017), Colonized strains may convert to infection by acquiring additional resistance genes through mechanisms such as horizontal gene transfer, gene mutation, and so on. In summary, the resistance rates in the infection group are significantly higher than those in the colonization group, indicating that we should enhance the monitoring and research of resistant strains in clinical practice. Particularly, preventing and controlling the spread of resistant bacteria will be an important focus for future research and practice.

In this study, we constructed Classification and Regression Trees (CART), C5.0, Gradient Boosting Machine (GBM), Support Vector Machine (SVM), Random Forest (RF), and Nomogram models through univariate logistic regression analysis. All six models achieved AUC values above 0.75, indicating that our research can provide a reference for differentiating between KP colonization and infection in clinical settings. The Random Forest model performed the best across all metrics, with an AUC of 0.99, an accuracy of 0.93, effectively distinguishing between KP colonization and infection status while significantly reducing the probability of misclassification. The precision was 0.98, indicating that nearly all samples predicted as infections were correct, thus reducing the occurrence of false positives. The Brier Score was 0.06, reflecting excellent predictive performance. In many practical scenarios, Random Forest has demonstrated outstanding performance, and this powerful ensemble learning algorithm is particularly well-suited for clinical data analysis and prediction (Jin et al., 2024; Mendapara, 2024; Wang Y. et al., 2024).

To better understand the relationship between the model and the data, we performed a visual analysis of the Random Forest model, which exhibited the best performance, by examining the importance of different factors using Mean Decrease Gini. These factors hold significant clinical relevance. Specifically, “Admitted with a diagnosis of respiratory infectious disease” is identified as the primary predictor of KP infection. The significant increase in infection risk for patients diagnosed with respiratory infectious diseases upon admission highlights the importance of early infection identification. This is particularly crucial as a large proportion of KP infections originate from the community, and studies have shown a strong correlation between KP-CAP (KP Community-Acquired Pneumonia) infections and higher, earlier mortality rates (Grosjean et al., 2024). This suggests that clinicians should promptly conduct pathogen detection and identification when seeing patients with respiratory infection symptoms, ensuring early detection, diagnosis, and treatment. Hypoalbuminemia, electrolyte imbalance, ESBL presence, and bedridden status are all important influencing factors for KP infection, closely related to the patient’s overall health status and immune function. Being bedridden indicates that the patient’s mobility is limited, which may lead to a series of health issues, such as muscle atrophy, venous thromboembolism, and complications like pneumonia. Hypoalbuminemia can result in malnutrition, reduced immunity, and poor healing capacity (Wang et al., 2023). This highlights the potential contribution of a patient’s nutritional and metabolic status to the risk of infection. For bedridden patients, it is crucial to regularly monitor serum albumin and electrolyte levels. An age greater than 62 years has been identified as an important risk factor, which is consistent with previously published studies indicating that nearly half of KP infections (45.7%) occur in the elderly population (Liu and Guo, 2019). This may be related to the decreased immune function and the prevalence of underlying diseases in the elderly population. The number of positive sputum cultures for KP (greater than 2) indicates a higher probability of infection, suggesting that multiple cultures are crucial for improving diagnostic accuracy in clinical practice. Invasive procedures such as tracheostomy and the placement of nasogastric tubes are also significant factors in KP infections. These invasive interventions can partially disrupt the normal structure of the upper respiratory tract, affecting the cleanliness and protective functions of the airway, making infections more likely. When the duration of mechanical ventilation exceeds 96 h, the risk of infection significantly increases. This may be related to the formation of respiratory biofilms, which can serve as breeding grounds for bacteria and elevate the risk of infection (Mishra et al., 2024). Craniotomy is also an important influencing factor, which may be because craniotomy is often difficult and lasting for a long time, and some patients are critically ill and need immediate surgery. Inadequate preoperative preparation and non-standard use of prophylactic antibiotics can lead to an increased probability of infection (He et al., 2024). Recent use of local corticosteroids can suppress local immune responses, reducing the body’s defenses against infections and making it easier for infections to develop. This immunosuppressive effect can lead to an increased risk of KP infections, particularly in patients who may already be vulnerable due to other underlying health conditions or invasive procedures (Fernández Peláez et al., 2000). Patients with disorders of consciousness usually lack the cough reflex to effectively clear respiratory secretions, and at the same time, the probability of aspiration is increased, which increases the risk of KP infection (Kobata, 2023).

Although the Random Forest model demonstrated good overall performance, it is not very intuitive. In contrast, a Nomogram is an intuitive visual tool used to display the risks or outcomes of multivariable prediction models. It is easy to use and understand, and it clearly illustrates the relative contributions of various factors to the outcome, making it particularly suitable for personalized decision-making in clinical practice (Yang et al., 2022; Gao et al., 2024; Luo et al., 2024). This study found that certain factors can serve as important criteria for assessing whether KP is colonized. When a patient’s sputum culture results indicate CRKP (Ceftazidime-Resistant KP), or if the patient has a nasogastric tube in place, is undergoing tracheostomy, or is in the ICU, we must exercise greater caution and thoroughness in assessment and judgment rather than blindly initiating antimicrobial therapy. Only by comprehensively considering the patient’s specific circumstances can clinicians develop more rational treatment plans, significantly improving the targeting and effectiveness of treatment while minimizing the misuse of medications and the emergence of resistance.

Although this study provides important insights into the diagnosis and management of KP infections, several limitations remain, which we hope to overcome in future research. First, the study included only 286 cases of KP positivity, and the data were sourced from a single center. Future studies should aim to expand the sample size and conduct multi-center collaborative research to enhance the generalizability and reliability of the findings. Moreover, the determination of colonization and infection relies on clinicians’ judgments and experiences, which may vary among different doctors and affect the accuracy of the results. We look forward to the development of more standardized clinical guidelines to reduce subjective differences among physicians and improve diagnostic consistency. Although this study identified 15 influencing factors, the occurrence of infections in clinical practice may be affected by other potential factors, such as the composition of the microbiome, environmental factors, and the psychological state of the patient. Additionally, molecular mechanisms, such as the development of bacterial resistance and the host’s immune response, are also important influencing factors. However, due to limitations in laboratory conditions and funding, we were unable to include these factors in this study. We hope that future research can develop a more comprehensive clinical model to provide clinicians with a more scientific and accurate basis for their judgments. Additionally, we anticipate integrating our constructed model into a clinical decision support system (CDSS) in the future. Regular evaluation of the model’s performance, along with the collection of feedback from healthcare professionals and patients, will be critical. This will allow us to update the data and retrain the model, continuously improving its usability and accuracy.

## 5 Conclusion

In this study, we successfully constructed six predictive models. Among them, the Random Forest model performed the best, achieving an area under the curve (AUC) of 0.99, with an accuracy of 0.93 and a precision of 0.98. This model effectively distinguishes between KP (KP) infection and colonization status, significantly reducing the rate of misjudgment. The Brier Score was 0.06, further validating the model’s excellent predictive performance. Key factors such as being diagnosed with respiratory infections upon admission and the number of positive sputum cultures underscore the importance of early diagnosis and repeated cultures. Additionally, factors closely related to the overall health status of patients, such as being bedridden, electrolyte imbalances, and hypoalbuminemia, significantly impact the risk of KP infection. To enhance the model’s applicability in clinical practice, we employed an intuitive nomogram, making it easier for clinicians to understand and utilize multivariable predictions. When faced with patients whose sputum cultures test positive for CRKP, or who have nasogastric tubes, tracheostomies, or are receiving ICU treatment, it is crucial to conduct a more cautious evaluation rather than initiating antimicrobial therapy blindly. This approach allows for more precise decision-making in patient management. We look forward to applying artificial intelligence technologies in future research to improve the accuracy of infection diagnoses, optimize antimicrobial treatment strategies, reduce unnecessary use of antibiotics, and thereby enhance patient outcomes and decrease the burden on healthcare systems.

## Data Availability

The original contributions presented in the study are included in the article/Supplementary material, further inquiries can be directed to the corresponding authors.
